# Height with Age Affects Body Mass Index (BMI) Assessment of Chronic Disease Risk

**DOI:** 10.3390/nu15214694

**Published:** 2023-11-06

**Authors:** Peter R. Holt, Osama Altayar, David H. Alpers

**Affiliations:** 1 Laboratory of Biochemical Genetics and Metabolism, Rockefeller University, New York, NY 10065, USA; holtp@rockefeller.edu; 2 Department of Medicine, Washington University School of Medicine, St. Louis, MO 63110, USA; osamaaltayar@wustl.edu

**Keywords:** BMI, age, obesity, height, disease risk

## Abstract

Body mass index (BMI) is a function of weight and height, but changing height has not been emphasized. Using the Framingham Heart Study with 5 decades of data on anthropomorphic measurements and disease states, changing height with age was extracted, and BMI was calculated using current and “young” height (calculated as height at age < 40 years). Decreased height began at age 40, with a mean loss from ages 40 to 80 of 4.8 cm for women and 3.6 cm for men. Using cutoff values of 25 and 30 for overweight and obesity, ~12.5% of women and ~10% of men were misclassified. Comparable figures for obesity classification were ~10 and 8%. At age 70, ~20% of women and ~15% of men were misclassified. Using the BMI corrected to “young” height, obese subjects had an increased risk for developing pre-diabetes and diabetes, with a higher risk for women than men. Using corrected BMI, obese subjects had a higher risk for developing hypertension, lower than for diabetes and higher for men than for women. These data do not establish whether the increased disease risk is clinically important but demonstrate that there is an advantage to using BMI corrected for “young” height when compared with BMI using current age-related height.

## 1. Introduction

In 1985, the NIH adopted the BMI as a “simple measurement highly correlated with other estimates of fatness” [1]. Even though this correlation has been extensively questioned, the simplicity of the measurement and the potential value of such an association for assessing disease risk for individual patients led to the widespread use of the BMI. The BMI probably misrepresents the risk of chronic diseases associated with obesity because it is not linked tightly with measurements of fatness, especially at higher values [2]. In the elderly, BMI is a suboptimal marker for adiposity since a dual X-ray absorptiometry (DEXA) scan measurements of fatness showed that a BMI over 30 had a low sensitivity and only moderately high specificity for obesity [3]. This lack of specificity resulted in difficulty in the interpretation of the BMI in the overweight range (BMI 25–27) and reaching any agreement on a cutoff value for overweight and obesity [4]. 

Neither height nor age are considered independent risk factors in calculating BMI, yet height decreases as age increases over the age of 50. Sorkin summarized the results of 16 studies that reported data for subjects up to 80 years of age, finding that from the age of 30 years, men lost an average of 5 cm and women 6 cm in height [5]. This loss in height could account for an average increase in BMI of ~1 kg/m^2^ by age 70. A weight reporting bias has been well recognized [6], but the height reporting bias did not change with time, which seems inconsistent with the documented loss in height beginning in the 4th decade. People appear unaware of their increasing loss of height with age. This failure to note a loss of height may be related to the fact that about half of the reduced stature of older individuals is a birth cohort effect, while the other half is an actual decrease in height after the age of 40 years [7].

We have reported that the measurement of the rate of change of anthropomorphic and physiological parameters is superior to current measurement values [8]. That study required the conversion of the Framingham Heart Study data into a more searchable format and allowed access to longitudinal height data covering 2–3 more decades than those reported by Sorkin in 1999 [9]. We postulated that utilizing maximal height might be a better proxy measurement for body build, which is known to correlate with fatness. The current study compares current BMI calculation with corrected ‘young’ BMI values and examines the association of cutoff values with two obesity-related diseases: type-2 diabetes and hypertension.

## 2. Methods

The use of the Framingham Heart Study (FHS) for data extraction and analysis has been reported by us previously using a publicly available database [8]. The study was submitted to the IRB of Rockefeller University and judged to be exempt from further review on 7 July 2023.

### 2.1. Participants

The FHS is a long-term population-based cohort study that aimed to identify the risk factors of cardiovascular diseases in the city of Framingham, Massachusetts, starting in 1948. The study recruited individuals aged 30 to 62 years with no history of cardiovascular diseases. In this study, we included participants who had at least one measurement of height before the age of 40 and at least one measurement after the age of 40. Each individual is compared to themselves over the period of follow-up. Height was measured with participants in their stocking feet using an anthropometer, and the measurements had excellent test–retest reliability [9].

### 2.2. Outcomes

The first outcome of interest was the magnitude of excess BMI resulting from height loss with aging. To assess excess BMI, we had to define the current BMI and a corrected BMI for each participant. The current BMI was calculated based on the current height and weight measurements for each participant, as it was recorded in the FHS data. The corrected BMI was defined as the BMI calculated using the average heights measured at ages ≤ 40 (the young height), instead of the current height, for each participant. Excess BMI was then calculated by subtracting the current BMI from the corrected BMI.

The second outcome of interest was the fraction of individuals that were misclassified as overweight (BMI 25–29.9) or obese (BMI ≥ 30) based on their current BMI compared to their corrected BMI. The third outcome of interest was to compare the correlation between the two BMI measurements and clinically important outcomes, pre-diabetes, diabetes, and hypertension. 

To determine whether the change in BMI classification using the current BMI vs. the corrected BMI in our study would demonstrate an effect on risk prediction for clinically important outcomes, we selected two measures (blood glucose and hypertension). Pre-diabetes was defined as non-fasting blood glucose > 140 mg/dL and diabetes as blood glucose > 200 mg/dL. An individual was considered to have hypertension if they were coded as taking an antihypertensive medication or if they had a systolic pressure ≥ 140 mmHg or diastolic pressure ≥ 90 mmHg on a study visit. Blood pressure measurements were the average of two physician readings, in some cases supplemented by additional nurse or technician readings.

### 2.3. Predictors

To allow us to calculate the corrected BMI, we needed to determine the height at different ages. In the FHS data, the height data contained clear outliers with growth or shrinkage of multiple inches. Additionally, the height measurement was rounded to the next lower quarter inch. This led to oscillating measurements for those near the rounding thresholds. To address these issues, locally weighted scatterplot smoothing (LOWESS) regression was performed to smooth the height-vs-age data for individuals with ≥4 data points (below which LOWESS cannot be used, Supplemental Figure S1), and also to perform outlier detection to identify and exclude data points particularly poorly fit by the regression line. Data points were excluded if they were more than six times farther from the LOWESS regression line than the median data point and were also at least 1 cm from the regression line; the regression was then recalculated without those points.

The BMI data were subject to similar outliers, and so LOWESS smoothing was also performed on these data (with outliers removed if they were more than six times farther from the LOWESS regression line than the median data point and were also at least 0.2 BMI units from the regression line). Weight was provided to the nearest 5 lbs. In the rare cases where the recorded BMI and that calculated from the recorded weight and height (smoothed height when available) differed by ≥5 BMI units, the BMI at those time points was excluded from analysis.

The young height of any individual was defined as the average of all height measurements conducted at age 40 or before (range 1 to 3 exams). Age-related shrinkage was then calculated as cm of height loss from the young height. When the LOWESS smoothed data were available (individuals with ≥4 data points), the young height and shrinkage were calculated from smoothed data.

The corrected BMI was then calculated using the current weight and young height, and we classified individuals into different groups based on their current and corrected BMI (Table 1). 

Table 2 summarizes the definitions used in this study. 

### 2.4. Statistical Analysis

The overall trends in height loss (age-related shrinkages) for males and females were described by linear and quadratic functions of height loss vs. years after age 35. Excess BMI related to age-related shrinkage was also modeled using a linear function.

The fractions of individuals who were misclassified as overweight and obese were plotted against age to show the impact of age-related shrinkage on misclassifying individuals as obese or overweight.

Calculation of the relative risk for blood glucose elevations in the obese vs. non-obese populations was performed using the current BMI or the corrected BMI to define obesity. The relative risk was calculated for both the current and corrected BMI as the fraction of the obese population divided by the comparable fraction of the non-obese population. The same calculation was performed for patients who received a diagnosis of hypertension. The relative risks were also plotted against age to show the impact of misclassification on the ability of BMI to predict clinically important outcomes.

The statistical analysis and plots were conducted and produced using Python 3.8.1 and the associated packages.

## 3. Results

Of the 5079 participants in the original FHS study, 1824 (814 male and 1010 female) had at least one height measurement before age 40 and at least one after age 40 and were included for analysis.

**Figure 1:**
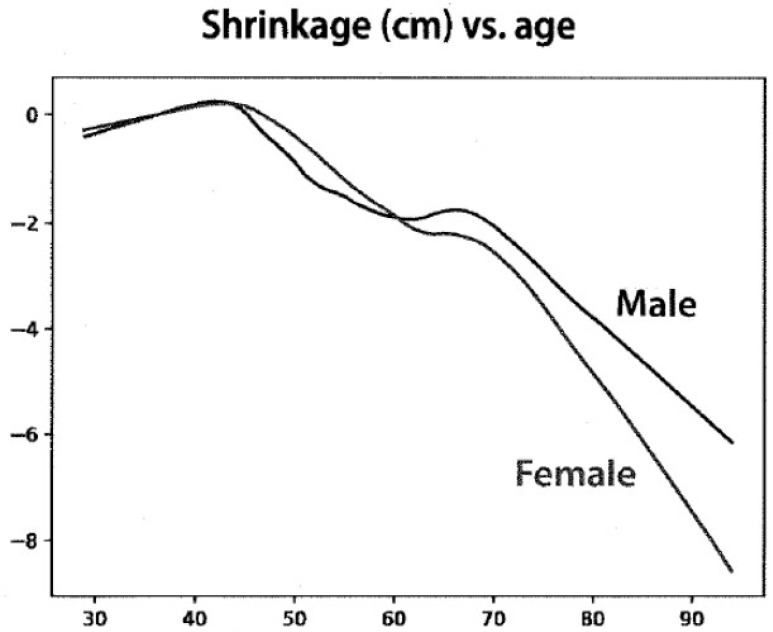
Linear fit of height observations from 1824 subjects selected from the Framingham Heart Study who enrolled before the age of 40 and who had at least 4 height measurements recorded. The X axis shows the age in years, and the Y axis shows the change in height in cm.

Age-related shrinkage (height loss) was modeled as linear and quadratic functions. Figure 1 shows the shrinkage with age for males and females using the linear function. Height loss from age 40 to 80 was calculated as 4.8 cm for women and 3.6 cm for men. Similar results were obtained when modeled as a quadratic function 

**Figure 2:**
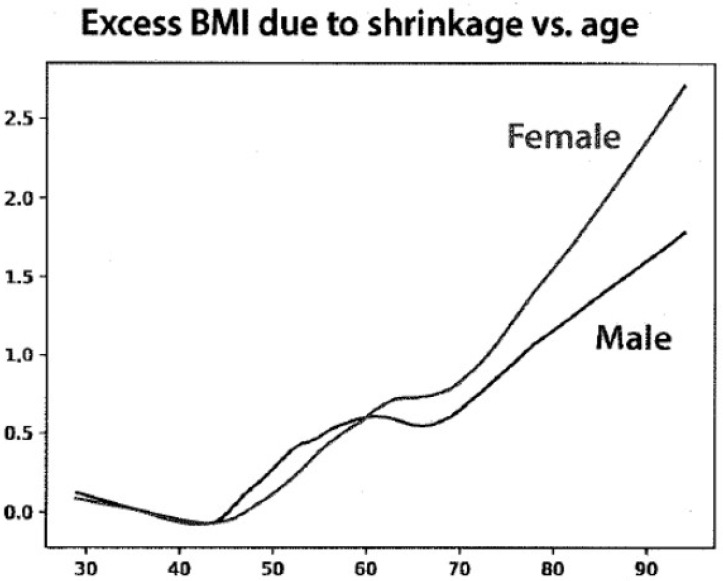
The absolute differences with age between body mass index (BMI) calculated from current height and height corrected to maximal height before age 40. The X axis shows the age in years, and the Y axis shows the excess BMI.

The young height data were then used to determine the excess BMI due to age-related shrinkage (Figure 2). The distribution of the current BMI ranged from 17 to 35 for women and from 19 to 35 for men. The changes in BMI units ranged from 0.5 to 2.5 between the ages of 70 and 90 years but were greater for women than for men.

**Figure 3:**
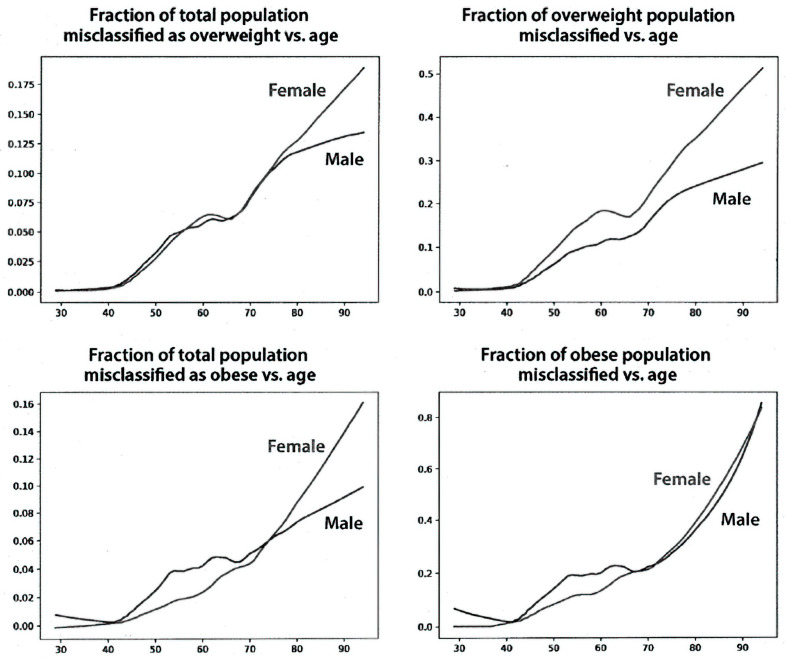
Fraction of study population misclassified as overweight or obese, determined as described in Methods. The X axis represents the age in years, and the Y axis represents the fractions misclassified.

Figure 3 shows the fraction of the study population with an incorrect BMI classification for the overweight and obese categories. Misclassification starts after age 40 for both men and women and increases linearly at least until age 80. Misclassification as overweight at age 80 reaches ~12.5% for women and ~10% for men for the entire population (left upper panel). Misclassification as obese reaches ~10% for women and ~8% for men (left lower panel). The fraction of misclassified overweight subjects at age 80 was ~40% for women and 20% for men (right upper panel). The fraction of misclassified obese subjects at age 80 was ~30% for both women and men (right lower panel). Using the corrected BMI for each individual subject, almost 20% of 70-year-old women and 15% of 70-year-old men were misclassified compared with the BMI calculated using their current height data. 

**Figure 4:**
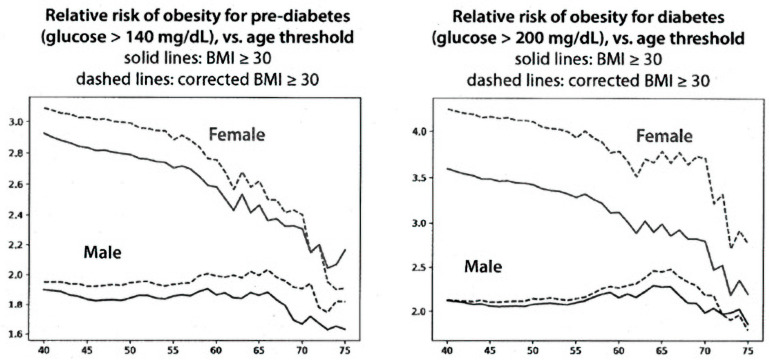
Relative risk of obesity for pre-diabetes and for diabetes using current and corrected BMIs. The X axis shows the age in years, and the Y axis shows the relative risk of obesity for diabetes and pre-diabetes.

Figure 4 (left panel) shows the relative risk of obesity for having pre-diabetes. Obese women at age 50 have about a 2.8-fold increased risk of having pre-diabetes compared to non-obese women. The dashed line shows that the risk is somewhat higher for women who are classified as obese, corrected by using their young height. The difference for males is similar, although the shape of the curve as a function of age differs. The right panel of Figure 4 shows the relative risk of obesity for having diabetes. The differences for diabetes in women are somewhat greater than for men for pre-diabetes. In all cases, however, the risk for elevated blood sugar at a given age is greater when the BMI is calculated using the height correction than it is using the current BMI. 

**Figure 5:**
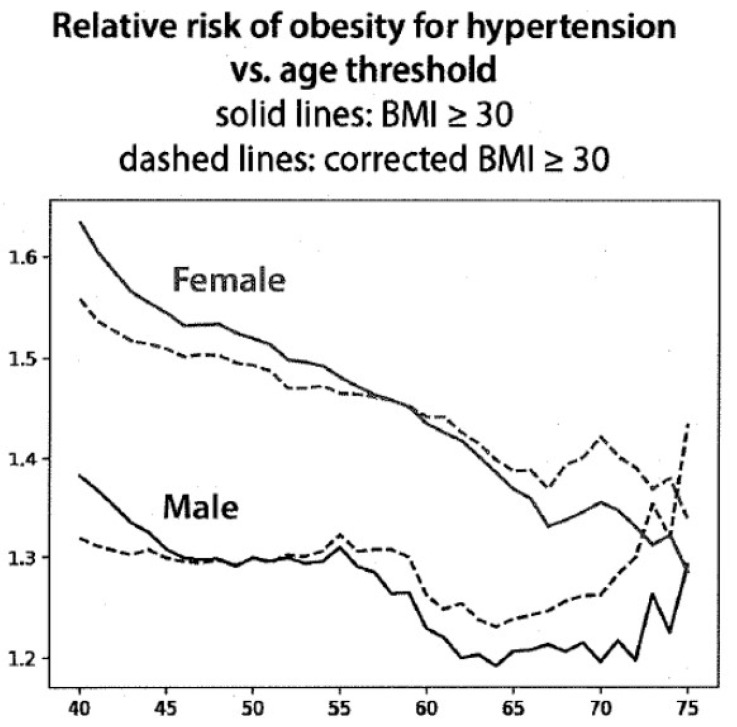
The risk of obesity for hypertension vs. age comparing current BMI to corrected BMI. The X axis shows the age in years, and the Y axis shows the relative risk of obesity for hypertension.

The correlation of hypertension as a function of age, using current and corrected BMI in males and females, is shown in Figure 5. After the age of 55 years for males and 60 for females, the relative risk of having hypertension using the corrected BMI was increased and progressively became greater with age, although the curves are moving differently for males and females. These differences, however, are consistent with the data for blood sugar shown in Figure 4. 

## 4. Discussion

The quest for reliable anthropomorphic markers to assess the risk of chronic disease has been going on for some time, but body height has been relatively overlooked. Body height is not fixed over the course of a lifetime. A decline begins at about age 30 and increases more rapidly after the age of 50 [10,11]. Women are at greater risk than men to decrease their height with aging, in part due to postmenopausal osteoporosis. Both genders develop narrowed intervertebral joint spaces and thoracic kyphosis. However, factors other than age affect height, including diet, genetics, and a relatively sedentary lifestyle. 

In the early 20th century, weight became an easily measured risk factor with the availability of home scales. The first population samples analyzed were insured subjects, but the values for weight and height often were self-reported and so were quite inaccurate. The Metropolitan Life tables included weight and height as risk factors for mortality but did not include the factor of age. Mortality risk at any given weight and height was stratified by an estimate of body frame (small, medium, or large) [12].

Subsequently, body types were divided into somatotypes (ectomorph, mesomorph, and endomorph) using height and weight measurements [13,14]. Factors that determined somatotypes included muscle mass, fatness, and skeletal structure for both genders and limb fatness for women [15]. Most body frame types were found to be relatively stable regardless of age [14]. The determination of somatotypes was complicated, relying on multiple anthropomorphic measurements and complex formulae.

The premise for Ancel Keys’ 1972 classic paper was that body weight in proportion to height “should indicate something about ‘build’ or shape and about obesity or fatness” [16]. He then showed that the Quetelet index (weight in kg/height in meters^2^) correlated better with obesity and fatness (assessed by skin-fold measurements) than did the “ponderal index” championed by Sheldon [13]. Keys appropriated the term ‘body mass index’ for the Quetelet index. This index was initially meant to define the average person in a population, but subsequently, it was applied to individual subjects. 

Problems with the derivation of BMI by Keys included the absence of age as a factor and the lack of consideration of body build. Keys’ study showed that only about half of the total variance in body fatness was accounted for by BMI but that this variance was not significantly related to height. Importantly, the age of the male populations that he studied did not extend beyond 60 years, so the decrease in height with increasing age was not appreciated. Keys stated that “the BMI was more stable than the ponderal index as height increased during growth and could be validated against other indices as weight increased to a maximum height”. Thus, variation in height was not subsequently included among independent risk factors that were associated with chronic disease. BMI only classified 55% of patients to their ‘correct’ somatotype [17].

Our data demonstrate the loss in height over a 40-year period between the ages of 40 and 80 in the Framingham Health Study. In the Baltimore Longitudinal Study of Aging, mean height loss for women and men between ages 40 and 80 were 7 cm and 6 cm, respectively [10]. Cross-sectional studies have described losses of height of as much as 10 percent of the “young height” [18]. These measurements of height loss are greater than our data, showing height losses of 4.8 cm and 3.6 cm, respectively, for women and men. These differences might be related to the cohort used in the current study, which included only those subjects that had been enrolled before the age of 40 years. However, the trends are very similar, and the change in BMI of between 1 and 2 units is the same as reported in a review of multiple studies by Sorkin in 1995 [5]. Any greater loss of height with age than we found would amplify the results of our study.

Self-reported height is generally over-estimated, while self-reported weight is under-estimated [19]. Loss of height alone has been shown to correlate with an increase in mortality [20]. One systematic review included studies of both measured and self-reported weight and height [21]. These data were confirmed in studies that used measured weight and height only, and the hazard ratios were higher in studies using self-reported weight and height. 

Many difficulties in measuring height in elderly persons have been recognized [21,22], and several methods have been suggested to overcome these issues. One of the simplest is the demi-span measurement, and because of its simplicity, it has been considered for use in estimating the height of elderly subjects. However, the measured height was less than the demi-span-estimated height in both men and women in a study from England [23], which led to a higher prevalence of overweight and obesity in women over the age of 65. Moreover, in a population of elderly Swedish people, the demi-span underestimated the percentage of the population classified as malnourished or obese in both genders when compared with the current BMI [24]. 

Others have noted similar changes in BMI with age. One study suggested that the modest change in BMI with age has no clinical consequence [25] since BMI correlated just as well with adiposity (measured by DEXA scan) after correcting for (self-reported) maximum height as it did without this correction. However, the corrected calculated BMI decreased by about 1–2 BMI units, similar to our findings. Moreover, the study by Heymsfield et al. [26] found that adiposity was independent of height, so adiposity may not be the best measure of the effect of altered BMI. 

A corrected BMI would be expected to be less than that based on current height, thus creating a lower risk for chronic disease (the opposite of our findings in Figure 4 and Figure 5). We use the term ‘risk’ for developing disease as the data report the presence of the two diseases examined during a specific time, succeeding decades. This use is similar to that in other epidemiologic studies. The finding of increased disease risk with the corrected BMI is probably due, in part, to the removal of subjects at lower risk who were included in the overweight and obese categories using current height data. The changes in BMI units reported here ranged from 0.5 to 2.5 between the ages of 70 and 90 years, and the distribution covered the range from 17 to 35 but was greater for women than for men. Despite this modest difference, the distribution covering the cutoff values for overweight and obesity might explain why such modest changes in BMI could have robust effects on the classification of risk by BMI since a large fraction of the population could have had a BMI near the cutoff values between 25 and 30, which are used to define overweight and obesity.

There are both strengths and weaknesses in the current study. The greatest strength is the availability of height data obtained by trained personnel over 5 decades in the Framingham Heart Study and the longitudinal follow-up of over 1800 subjects. However, in order to determine a “young” height before the age of 40 years, only a relatively small cohort could be included in the analysis. Nevertheless, this cohort reproduced the age-related height shrinkage previously reported by others. One weakness of the study is that the FHS data were derived primarily from a Caucasian population, similar to the Baltimore Aging study [10], so it might not apply to other ethnic groups. Future studies will be needed to confirm these data in other populations. Our data also are not sufficiently large for us to analyze the effect of a corrected BMI on many important age- and BMI-related risks, such as for cardiovascular and cancer diseases. 

BMI will continue to be used to define obesity despite its lack of precision in detecting adiposity and its exclusion of the effects of age and body build. Available data demonstrate that the risk of chronic disease increases with age. However, it is still unclear how large an effect the misclassified BMI, caused by the age-related decrease in height, might have on disease risk.

## Figures and Tables

**Table 1:** BMI categories based on current and corrected BMI.

Category	Current BMI	Corrected BMI
Obese	≥30 kg/m^2^	≥30 kg/m^2^
Wrong obese	≥30 kg/m^2^	<30 kg/m^2^
Overweight	25–29.9 kg/m^2^	25–29.9 kg/m^2^
Wrong overweight	25–29.9 kg/m^2^	<25 kg/m^2^

**Table 2:** Definitions used in the study.

Current Weight	Weight in kilograms at the current age
Current height	Height in centimeters at the current age
Young height	The average of all height measurements conducted at age 40 or before
Age-related shrinkage (height loss)	The amount of height lost in cm from the young height
Current BMI	Body mass index calculated using current weight and current height
Corrected BMI	Body mass index calculated using current weight and young height
Excess BMI	The difference between current BMI and corrected BMI

## Data Availability

The raw data for the Framingham Heart Study are available at https://biolincc.nhlbi.nih.gov/studies/framcohort/ (Accessed on 5 October 2023).

## Supplementary Materials

The following supporting information can be downloaded at: https://www.mdpi.com/article/10.3390/nu15214694/s1, Figure S1: The upper panel is an example of the use of a locally weighted scatterplot individual with a small number of observations that precluded the use of LOWESS. The blue line represents the values as reported by the Framingham Heart Study, and the orange line represents the values estimated using LOWESS regression. The X axis shows the age in years and the Y axis shows the height in meters.
