# Supplementary material for: Height with Age Affects Body Mass Index (BMI) Assessment of Chronic Disease Risk
Source: Nutrients. 2023 Nov 6;15(21):4694. doi: 10.3390/nu15214694 (PMC10649148; doi:10.3390/nu15214694)
Supplement: Supplementary file 1 [file nutrients-15-04694-s001.zip › nutrients-2666061-supplementary.pdf]

Supplemental data:

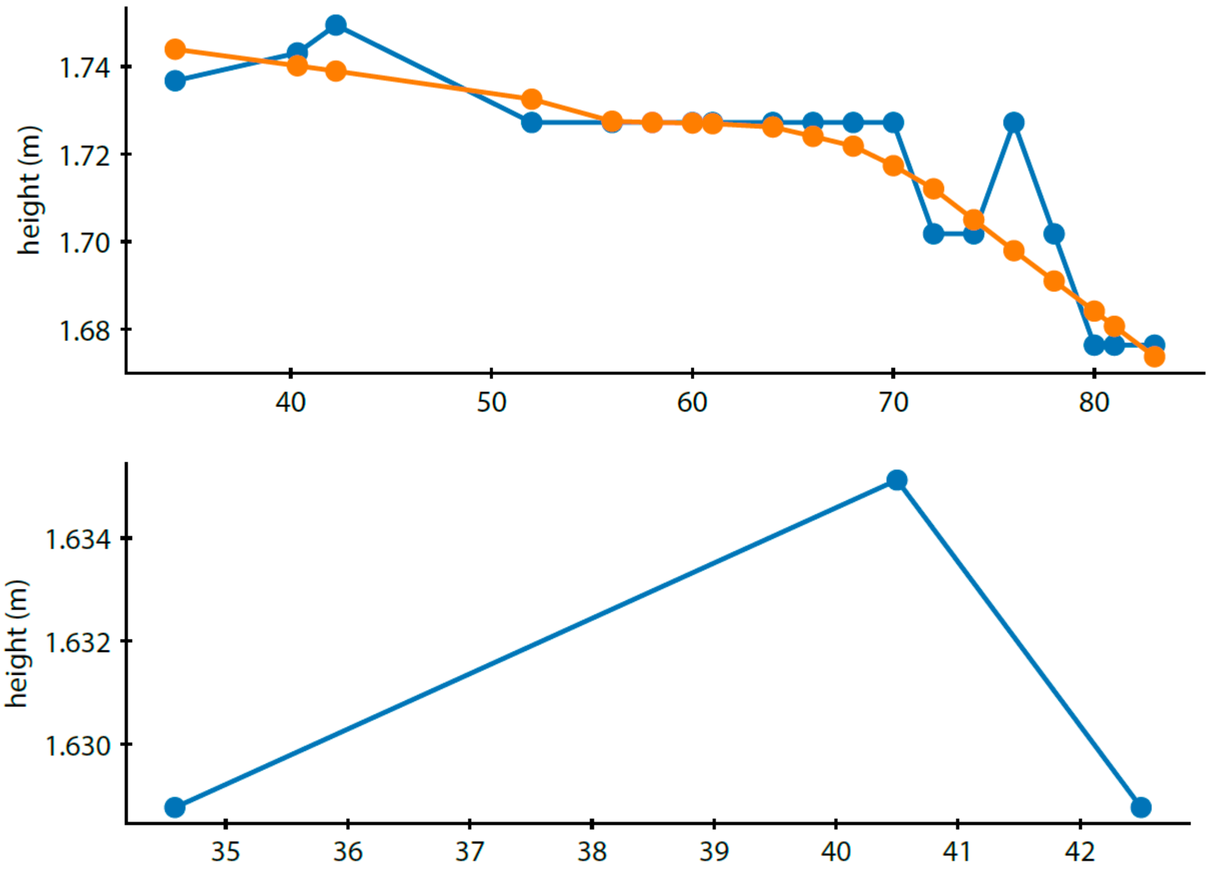

Supplemental Figure S1. The upper panel is an example of the use of locally weighted scatterplot individual with small number of observations that precluded the use of LOWESS. The blue line represents the values as reported by Framingham Heart Study, and the orange line represents the values estimated using LOWESS regression. The X axis shows the age in years and the Y axis height in meters.
